# Nasal nalbuphine analgesia in prehospital trauma managed by first-responder personnel on ski slopes in Switzerland: an observational cohort study

**DOI:** 10.1186/s13049-021-00852-y

**Published:** 2021-02-17

**Authors:** Urs Pietsch, Yoël Berger, David Schurter, Lorenz Theiler, Volker Wenzel, Lorenz Meuli, Andreas Grünenfelder, Roland Albrecht

**Affiliations:** 1 Department of Anaesthesiology and Intensive Care Medicine, Cantonal Hospital St. Gallen, Rorschacher Strasse 95, 9007 St. Gallen, Switzerland; 2 Swiss Air-Ambulance, Rega (Rettungsflugwacht / Guarde Aérienne), Zürich, Switzerland; 3 Department of Emergency Medicine, Inselspital, Bern University Hospital, University of Bern, Freiburgstrasse, 3010 Bern, Switzerland; 4 Department of Anaesthesiology and Pain Medicine, Inselspital, Bern University Hospital, University of Bern, Bern, Switzerland; 5 Schutz & Rettung Zürich, Zürich, Switzerland; 6 Department of Anaesthesiology, Cantonal Hospital Aarau, Aarau, Switzerland; 7 Department of Anaesthesiology and Intensive Care Medicine, Friedrichshafen Regional Hospital, Friedrichshafen, Germany; 8 Department of Vascular Surgery, University Hospital Zürich, Zürich, Switzerland; 9 Department of Anaesthesiology, Klinik Gut, St. Moritz, Switzerland

**Keywords:** Nalbuphine, Prehospital analgesia, Trauma, Emergency medicine, Pain, Wilderness medicine

## Abstract

**Background:**

Pain is one of the major symptoms complained about by patients in the prehospital setting, especially in the case of trauma. When there is mountainous topography, as in Switzerland, there may be a time delay between injury and arrival of professional rescuers, in particular on ski slopes. Administration of a safe opioid by first responders may improve overall treatment. We therefore assessed administration of nasal nalbuphine as an analgesic treatment for trauma patients in Switzerland.

**Methods:**

This observational cohort study examined 267 patients who were treated with nasal nalbuphine by first responders in six ski resorts in Switzerland. All first responders were instructed to begin treatment by assessing the feasibility of using nalbuphine to treat pain in the patient. A treatment algorithm was developed and distributed to assure that nalbuphine was only administered following a strict protocol. Data regarding pain scores and pain reduction after administration of nalbuphine were collected on-site. Refills were handed out to the first responders with the return of each completed questionnaire.

**Results:**

Nalbuphine provided effective pain relief, with the median level of pain on the numeric rating scale for pain reduced by 3 units on average, from 8 points (*p* < 0.001). The multivariate regression model showed that pain reduction was more pronounced in patients with higher initial pain levels. Nalbuphine was more effective in adolsecents than in patients aged 20 to 60 years (*p* = 0.006). No major side effects were observed.

**Conclusion:**

Nasal administration of nalbuphine by first responders is a presumably safe and effective noninvasive pain management strategy for acutely injured patients in the prehospital setting. This may be an alternative, especially in the case of severe pain and prolonged time between arrival of the first responders and arrival of EMS/HEMS personnel on scene.

## Introduction

Pain is one of the major prehospital symptoms and requires prompt management, in particular in trauma patients. Nevertheless, many colleagues report that analgesia in the field is insufficient for multiple reasons, one being the overly long response times of the emergency medical service (EMS) [1–5]. In Switzerland, EMS units are staffed with ALS-qualified paramedics, but due to mountainous and alpine terrain, the time until arrival at the scene can be prolonged [6–9]. Therefore, Switzerland also has a long history of helicopter emergency services (HEMS), staffed with a pilot, a paramedic and an ALS-qualified emergency physician. Usually, a given HEMS service can reach every point in Switzerland within 15 min, subject to weather conditions [10].

Winter sports such as downhill skiing and snowboarding attract > 2.5 million skiers annually in Switzerland, with about 76,000 injured skiers requiring treatment [11]. First responders in an accident on a ski slope are usually ski lift employees who provide first aid but no analgesia. However, providing analgesia on ski slopes may be achievable with nalbuphine due to its safety features (mixed agonist/antagonist), its ceiling effect regarding respiratory depression, an analgesic potency approximately 0.8 to 0.9 times that of morphine [12], its simple handling and legal approval, its lack of potential for abuse, its safety in pregnant and lactating women, and its potential for use by first responders [13–16, 13, 17, 18].

There are several approved routes of nalbuphine administration, including intravenous (IV), intramuscular (IM), subcutaneous (SC) [13], and nasal, due to nalbuphine’s high lipophilicity and low molecular weight [17, 19–21, 22–24]. Because of its simplicity and non-invasive nature, we chose the nasal route for nalbuphine administration by ski slope first responders. Our hypothesis was that nasal nalbuphine would have no effect on pain in victims of ski slope accidents.

## Methods

This observational cohort study examined data collected from patients given nalbuphine analgesia by first responders in the prehospital phase. Reporting of the study conforms to the STROBE statement for the reporting of observational cohort studies.

### Study design

**Fig. 1:**
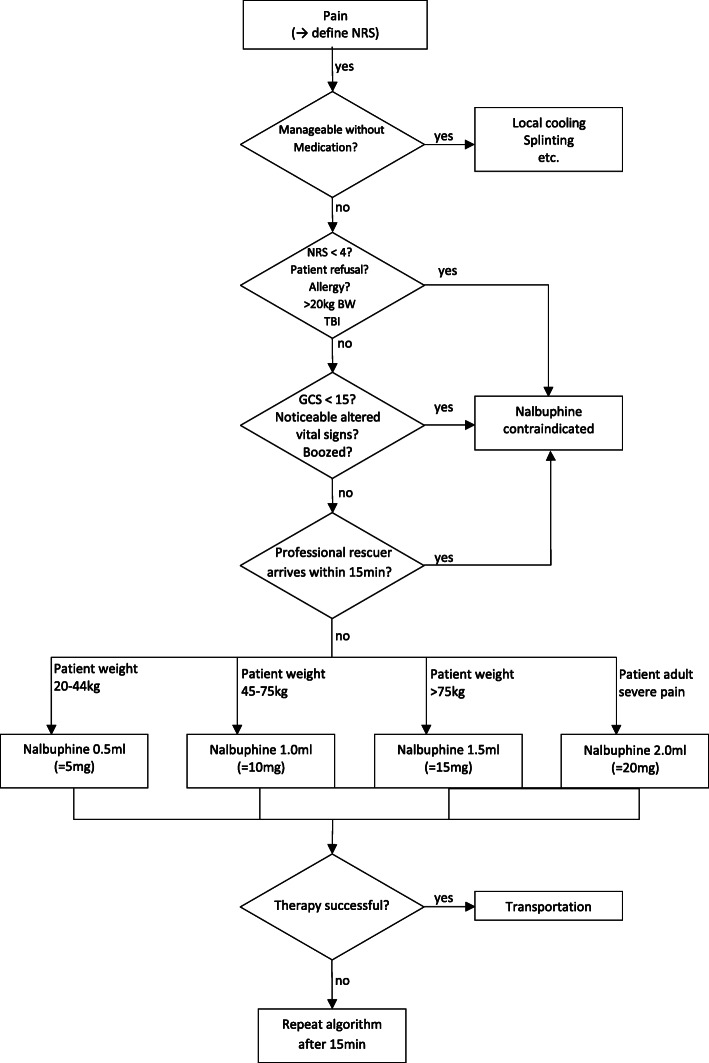
Treatment algorithm that had to be followed by the lay rescuers to assure the drug was administered only if indicated. NRS = numeric rating scale. GCS = Glasgow coma scale. Noticeable altered vital signs = Airway – threatened, respiratory rate > 36 or < 8 or SpO_2_ < 90%, Circulation - pulse > 140 or < 40, systolic Bloodpressure < 90 or > 180

This analytical observational cohort study was performed in six different ski resorts in the canton of Graubuenden, Switzerland (Arosa, Jakobshorn Davos, Lenzerheide, Marguns Engadin, Parsenn, and Weisse Arena Laax). All interested first responders received mandatory theoretical and practical instruction about nasal nalbuphine using the Mucosal Atomization Device (Teleflex, Wayne, PA, USA). Specifically, they were instructed about the mechanisms of action, indications and contraindications, as well as the potential side effects. Administration of nalbuphine according to an algorithm was required to assure patient safety (Fig. 1). First responders were free to choose to participate and to enrol patients. Therefore, the inclusion of patients was non-consecutive and there was no control group in this study. We report results from experience with nasal administration of nalbuphine from November 2017 until April 2020.

### Medication, administration, indications and contraindications

Nalbuphine (Nalbuphin OrPha®, OrPha Swiss, Kuesnacht, Switzerland) in a dose of 10 mg/ml in vials containing 2 ml (20 mg) was used. The dosage was according to body weight, with a minimum dose of 5 mg for patients of 20-44 kg up to a maximum dose of 20 mg for adults > 75 kg with severe pain (Fig. 1). The total dosage was divided in half to administer a maximum of 1 ml in each nostril. The main indication for nalbuphine was severe pain, defined as a score of ≥5 on a numeric rating scale (NRS), with 0 defined as absence of pain and 10 being the maximum imaginable pain. If there was evidence of altered consciousness, alcohol consumption or noticeable abnormal vital signs, administration of nalbuphine by first responders was deemed contraindicated. Head trauma was an absolute contraindication for nalbuphine regardless of changes in mental status, to assure that no nasal drug was applied as long as a skull fracture was not excluded. Further nalbuphine contraindications were known allergy to the drug or its additives, patient refusal, or body weight < 20 kg. Nalbuphine was also restricted to cases in which it was highly likely that the waiting time until arrival of an EMS or HEMS team was greater than 15 min.

### Data collection and statistical analysis

Data collection was performed directly by first responders using an online questionnaire with predefined endpoints (First responders online questionnaire in German; https://docs.google.com/forms/d/e/1FAIpQLScuvbf0s-n9Ev5EGcO10d9Vw1Gv9JHptJvV1smNeJZy-pTJLA/viewform). Apart from age and sex, no further personal data were recorded in order to ensure de-identification of patients. Also, no personal data of the first responders on scene were collected, so no conclusions about the rescuers could be drawn during later analysis.

### Statistical analysis

Patients’ characteristics were summarized and presented in tables. Continuous variables were summarized by mean ± standard deviation if normally distributed or by median and the interquartile range if skewed. Normality was tested using the Shapiro-Wilk test. Categorical variables were summarized with counts and percentages for each level of the variable. The Wilcoxon-Mann-Whitney-*U* Test was used to assess differences in pre- vs. post-nalbuphine NRS. To further elaborate on factors that are potentially associated with the effectiveness of nalbuphine, a multiple linear regression model was built including the variables sex, age in categories and initial pain level in a complete-case analysis. Due to heteroscedasticity of pain reduction depending on the initial reported pain level, the same regression model was built on a calculated relative pain reduction variable, i.e., percentage of NRS from initial NRS. Dose of nalbuphine applied was excluded from the model due to multicollinearity of this variable, and the initial pain level. Location of injury was not included in the model due to the low number of patients per injury location, and the substantial proportion of missing data for this variable.

## Results

### Patient characteristics

During the observation period, a total of 267 patients (male sex, 58.8%; age, 33.3 ± 18.2 years) were treated with nasal nalbuphine by first responders (Table 1). The most common part of the body injured were the upper extremities (44.2%), with more than half of these involving the shoulder (23.6% of all injuries). Lower extremities were injured in 35.2%, with a majority of knee and lower leg injuries (12 and 14.6% of total injuries, respectively). The remaining 20.6% were injuries of the trunk, thorax and abdomen. For 28 patients (10.5%) no data were available about the site of injury (Table 1).

**Table 1:** Baseline Characteristics and pain

Variable	Total ***n*** = 267
Age in years	
Mean, *± SD*	33.3 ± 18.2
< 20, *n (%)*	84 (31.5)
20–60, *n (%)*	163 (61.1)
≥ 60, *n (%)*	20 (7.5)
Male sex, *n (%)*	157 (58.8)
Pain (NRS)	
Initial, *median (IQR)*	8 (7 to 9)
After nalbuphine	5 (4 to 7)
Missing, *n (%)*	19 (7.1)
Pain reduction (NRS)	
Median (IQR)	−3 (−4 to −1)
Clinically relevant pain reduction, *n (%)*	145 (58.5)
Missing, *n (%)*	19 (7.1)
Dose of Nalbuphine	
5 mg, *n (%)*	24 (9.0)
10 mg, *n (%)*	128 (47.9)
15 mg, *n (%)*	35 (13.1)
20 mg, *n (%)*	80 (30.0)
Location of Trauma	
Shoulder, *n (%)*	63 (23.6)
Upper arm, *n (%)*	22 (8.2)
Elbow, *n (%)*	1 (0.4)
Forearm or hand, *n (%)*	32 (12.0)
Hip or femur, *n (%)*	22 (8.2)
Knee, *n (%)*	32 (12.0)
Lower leg, *n (%)*	39 (14.6)
Foot, *n (%)*	1 (0.4)
Neck, *n (%)*	0 (0.0)
Back, *n (%)*	8 (3.0)
Thorax or clavicula, *n (%)*	18 (6.7)
Abdomen, *n (%)*	1 (0.4)
Missing, *n (%)*	28 (10.5)
Adverse Events	
None, *n (%)*	252 (94.4)
Nausea or vomiting, *n (%)*	3 (1.1)
Retching, *n (%)*	5 (1.9)
Nasal discomfort, *n (%)*	3 (1.1)
Major adverse events, *n (%)*	0 (0)
Technical device problem, *n (%)*	4 (1.5)

Footnote:

No missing data if not stated explicitly. *SD* Standard Deviation, *IQR* Inter Quartile Range

**Fig. 2:**
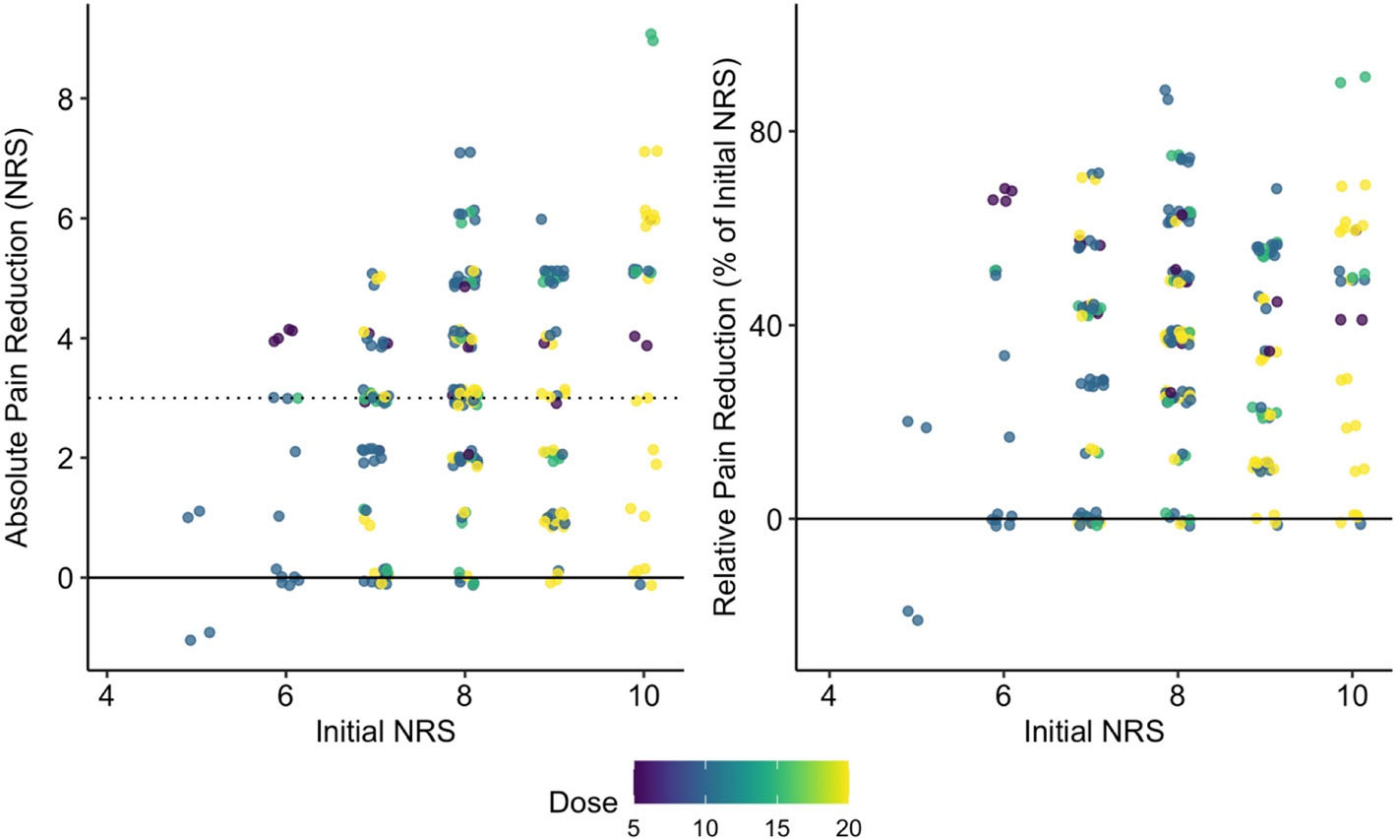
Distribution of pain reduction depending on initial pain level and dose applied. Footnote: To increase readability of the plot, the points were jittered around the true value. One hundred forty five patients had pain reduction of at least 3 units on the numeric rating scale. Absolute pain reduction in NRS points as reported by the trauma victims (left panel). The dotted line indicates the median pain reduction of 3 NRS units. Due to heteroscedasticity in pain reduction depending on the initial pain level, relative pain reduction was calculated and is presented as a percentage scale (right panel)

**Fig. 3:**
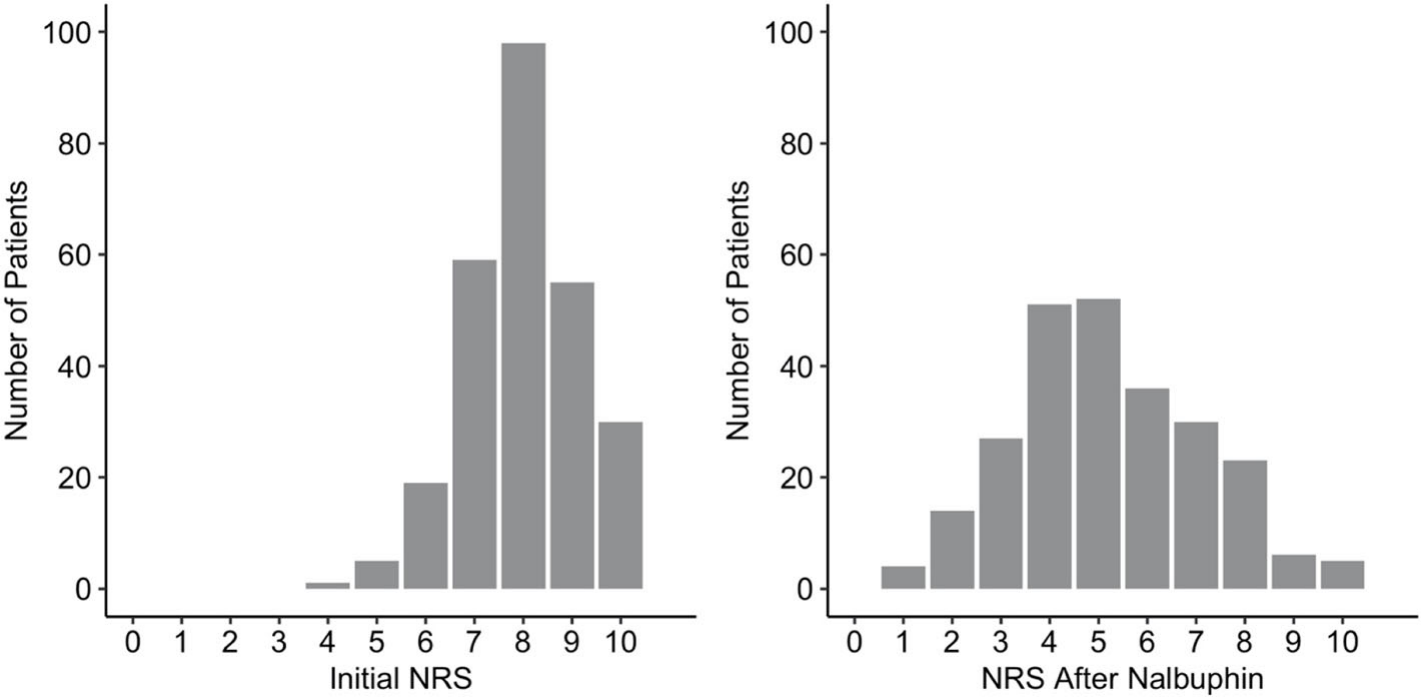
Initial pain level (NRS) versus pain level after administration of nalbuphine. Footnote: The initial pain level was reported in all patients. Information on pain level after administration of nalbuphine was missing in 19 of 267 patients. The difference in pain level was significant, *p* < 0.001 (Wilcoxon signed-rank test)

The trauma victims reported a median pain level of 8 points (IQR 7 to 9) on the numeric rating scale at the initial assessment by first responders. Nalbuphine caused a statistically significant and clinically relevant decrease in the level of pain, by a median of 3 NRS units, (*p* < 0.001) (Table 1, Figs. 2 & 3). The multivariate regression model showed that pain reduction was more pronounced in patients with higher initial pain levels. This effect decreased but remained statistically significant in the second multivariate model using a relative scale for pain reduction and thereby avoiding heteroscedasticity in the model (Table 2). Nalbuphine was more effective in adolsecents than in patients aged 20 to 60 years (*p* = 0.006). The same tendency was observed for the elderly population, (≥60 years of age); however, there were only 20 patients in this age group and the difference was not statistically significant. The level of pain reduction was similar for both sexes (Table 2). Forty-one patients (15.3%), expressed dissatisfaction with the treatment.

**Table 2:** Multivariate Linear Regression Models on Pain Reduction after Nasal Nalbuphine

Variable	Absolute NRS reduction model			Relative NRS reduction model (%)		
	Estimate	95% C.I.	*p*-value	Estimate	95% C.I.	*p*-value
Age < 20 years	0.0			0.0		
Age 20–60 years	−0.61	−1.11 to −0.10	0.018	− 0.87	−14.89 to −2.48	0.006
Age > 60 years	−0.59	− 1.54 to 0.36	0.221	−7.77	− 19.47 to 3.94	0.193
Male sex	0.0			0.0		
Female sex	0.08	−0.39 to 0.54	0.754	1.16	- 4.21 to 7.35	0.594
Initial pain (NRS)	0.51	0.31 to 0.72	< 0.001	2.53	0.03 to 5.04	0.048

Complete-case analysis: Included observations *n* = 244, 19 missing observations. Estimate = Difference in NRS reduction after application of nasal Nalbuphine according to either level of the factor variable or initial pain level (absolute difference or relative difference in percentage respectively). 95% C.I. = 95% confidence interval of the estimate. Male sex and Age < 20 years served as the reference groups in both models

Mild side effects were reported in 5.6% (*n* = 11) of patients. No major adverse events like intoxications which needed to be reversed by the intravenous administration of naloxon were observed.

## Discussion

In our study, the majority of the patients reported a mean reduction of 3 points on the NRS, without any major adverse events, after nalbuphine administration.

The pain relief reported in our study is consistent with the literature, where a decrease of more than 2 points on the NRS was deemed good pain relief [25]. A study with out-of-hospital use of nasal fentanyl also showed an average reduction of 3 points on the NRS, leading to the conclusion that nasal fentanyl is effective for analgesia in the pre-hospital phase [26].

Only a few studies have evaluated analgesia with nalbuphine in the prehospital setting, and they had conflicting results. When given by paramedics out-of-hospital, IV or IM nalbuphine reduced pain by 5 points on the NRS [27], while the same strategy resulted in excessive morphine requirements after hospital admission in a case series. That study even reported less decline in pain scores after further administration of analgesics in the emergency department in prehospital nalbuphine patients [28], which is in accordance with a case review that described increased opioid requirements after nalbuphine [18]. In fact, this is expected because of the mixed agonist/antagonist effects of nalbuphine, with agonistic effects on the κ-receptors and antagonistic effects on the μ-receptor [12]. Therefore, if analgesia is continued with pure μ-receptor agonists, an increased dose is needed to overcome the antagonistic effect exerted by nalbuphine. Another possible disadvantage of mixed opioid agonist/antagonists is a limited analgesic effect or ceiling effect [12]. This could explain why no patient was completely free of pain after nalbuphine, with a minimum NRS of one after treatment. We did not find any significant differences between sexes regarding pain intensity before or after nalbuphine, regardless of the administered dose. There is some evidence that nalbuphine has a pain-facilitating effect in males, at least if it is administered in small doses of 5 mg [29], an effect we did not observe. There was a predominance of males in our study (58.8% vs. 41.2%) which is consistent with the average distribution of injured people on Swiss ski slopes (56.6% male vs. 43.4% female) [30]. We observed only a few minor side effects in our patients. Although unlikely, the risk of respiratory depression, monitored by pulse oximetry and respiratory rate, also exists following nalbuphine [12, 16, 17], but this was not reported in our study. Interestingly, in our study only 15% of trauma patients reported dissatisfaction with nalbuphine, and though the number of patients who reported a good analgesic effect was high (60.0%). This phenomenon was already described in labouring women treated with nalbuphine: only 54–57% of the parturient women experienced good pain relief, but 78% were willing to have the same treatment in a subsequent birth [15]. This could possibly be explained by a reported feeling of relaxation, rather than pain relief [15].

There is also evidence that satisfaction with opioid treatment seems to be influenced, at least in part, by rapid onset [16]. Nasally administered drugs are normally well absorbed, with rapid onset of action due to the rich blood supply and large surface of the mucosa, bypassing of a first-pass-effect and, maybe at least in part bypassing the blood-brain-barrier [21].

Our study has some limitations. First, we could not follow up patients after handover to EMS/HEMS personnel. Second, we mainly observed traumatic shoulder and knee injuries, which is expected with the predominance of our study sites being in Swiss ski resorts [30]. Third, there could be a bias in data collection because it was performed directly by first responders. If a patient felt sympathies for his rescuer, it is possible that the answers have been whitewashed, in particular regarding qualitative outcomes such as overall satisfaction. Fourth, although the qualitative pain reduction was reported for each patient, there were missing NRS values after nalbuphine in 10.5% (*n* = 28) of the patients, which is to be expected in an observational study in the pre-hospital setting. There was also a predominance of the male sex, which could have had an influence on the described pain reduction. We observed only a few minor side effects with nalbuphine. However, we did not follow up on the patients, and we cannot prove that nalbuphine administration is always safe.

In conclusion, nasal nalbuphine administered by first responders was a noninvasive pain management strategy that provided effective and presumably safe analgesia in prehospital, acutely injured patients in our studied environment. This method may provide an alternative, especially in situations involving severe pain and prolonged time between arrival of the first responders and arrival of EMS/HEMS personnel on scene. This conclusion is viable in the setting described, involving trained first responders. Further studies would be needed to include an overall recommendation for laypersons.

## Data Availability

Not applicable.

## Abbreviations

CI: Confidence interval
CNS: Central nervous system
Da: Dalton
EMS: Emergency medical service, ambulance service
HEMS: Helicopter emergency medical service
i.m.: Intramuscular
i.v.: Intravenous
NRS: Numeric rating scale
s.c.: Subcutaneous
SD: Standard deviation
